# City Locomotion

**Published:** 1873-09

**Authors:** 


### CITY LOCOMOTION.

In all large and flourishing cities there must be expeditious and inexpensive methods of conveyance from one point to another. New York introduced street cars twenty years before Philadelphia, less than a hundred miles away, could be persuaded to adopt them. And, later still, George Francis Train obtained permission of the London authorities to lay down a track for a short distance, as an experiment, but the popular prejudice was so great that the "powers that be" annulled the grant, to the unmitigated disgust of the great Irrepressible. But now London has her tramways, and so has Paris; and, in fact, they are becoming so common that a town two rods long and two rods broad, if it can have a "horse car," feels as big as a boy the first day he sports a pair of suspenders or wears a long-tailed coat.

The first physiological horse car has yet to be built. Those in London have two advantages: there is either no window in front, or, if there is, it is immovable—thus effectually preventing some booby of a passenger from hoisting the window because he is too warm, thus endangering the health of a dozen others, who, having been sitting some time, are quite cool enough, or are already chilly. Many a person has been thrown into dangerous and even fatal illness, in New York, by having to sit in an omnibus or car with the forward windows and doors wide open. Another advantage of the London omnibus is that none of the side windows are movable—thus preventing one person from inconveniencing a dozen others, especially the one sitting next him, by opening the window, because at the moment of his entering the air seems "close" to him—almost suffocating—but if he will only wait a minute or two this sensation will pass away.

It is true, that on a warm day the air of an omnibus containing a dozen people is very close and almost stifling, but this great fact should be always borne in mind—a "faint" is the most that can happen in a too close or warm atmosphere, the person will always "come to" within say five minutes, and straightway be as well as ever; but a cold wind on an old person, or on a child or invalid, will, in less than five minutes, cause an uncomfortable feeling of cold, very liable to be followed by a chill, inducing dangerous affections of the lungs or other internal organs, and which, even if recovered from at all, is only done after weeks and months of painful suffering. Besides, if a vehicle is too close for one coming in, it would be better for him to leave it, and go elsewhere, than to endanger the health and life of a dozen others, who, by previous occupancy, have, of right, a preference to all the privileges of the vehicle. The London omnibus has ventilating apertures which cannot be closed, and, in addition, admit the air above the heads of the occupants. It is here suggested that every opening for ventilation should be covered with a wire grating, sieve-like, so that the air should come in so finely divided that it could not affect the sitter injuriously. Inattention to these items has occasioned the loss of health and life many times.

